# Supplementary material for: Comparative multiomics analysis of cell physiological state after culture in a basket bioreactor
Source: Sci Rep. 2022 Nov 23;12:20161. doi: 10.1038/s41598-022-24687-4 (PMC9686226; doi:10.1038/s41598-022-24687-4)
Supplement: Supplementary file 1 — Supplementary Information 1. [file 41598_2022_24687_MOESM1_ESM.zip › raw data/Transcriptomic raw data/6.Enrichment/1.GO/TE1vsTC1/GSEA/TC1/ANTIGEN_PROCESSING_AND_PRESENTATION/ANTIGEN_PROCESSING_AND_PRESENTATION(GO_0019882).html]

Details for gene set ANTIGEN\_PROCESSING\_AND\_PRESENTATION(GO:0019882)[GSEA]


|  || Dataset | readcount.sample |
| Phenotype | sample.cls |
| Upregulated in class | TC1 |
| GeneSet | ANTIGEN\_PROCESSING\_AND\_PRESENTATION(GO:0019882) |
| Enrichment Score (ES) | -0.61677647 |
| Normalized Enrichment Score (NES) | -1.3560421 |
| Nominal p-value | 0.0 |
| FDR q-value | 0.18902451 |
| FWER p-Value | 0.174 |
Table: GSEA Results Summary

  

Fig 1: Enrichment plot: ANTIGEN\_PROCESSING\_AND\_PRESENTATION(GO:0019882)
  
Profile of the Running ES Score & Positions of GeneSet Members on the Rank Ordered List

  

| PROBE | DESCRIPTION   (from dataset) | GENE SYMBOL | GENE\_TITLE | RANK IN GENE LIST | RANK METRIC SCORE | RUNNING ES | CORE ENRICHMENT || 1 | 103221695 | MHC-DOB |  |  | 85 | Infinity | -0.0027 | No |
| 2 | 103222360 | LOC103222360 |  |  | 13831 | 0.779 | -0.5287 | Yes |
| 3 | 119618080 | LOC119618080 |  |  | 15329 | 0.687 | -0.5179 | Yes |
| 4 | 103221688 | MHC-DMA |  |  | 15400 | 0.682 | -0.4439 | Yes |
| 5 | 103221837 | LOC103221837 |  |  | 15867 | 0.649 | -0.3913 | Yes |
| 6 | 103221771 | LOC103221771 |  |  | 16097 | 0.635 | -0.3297 | Yes |
| 7 | 103221700 | LOC103221700 |  |  | 17110 | 0.564 | -0.3111 | Yes |
| 8 | 119618283 | LOC119618283 |  |  | 17424 | 0.539 | -0.2641 | Yes |
| 9 | 103221786 | LOC103221786 |  |  | 17526 | 0.531 | -0.2085 | Yes |
| 10 | 119622860 | LOC119622860 |  |  | 18146 | 0.475 | -0.1824 | Yes |
| 11 | 103221775 | LOC103221775 |  |  | 18330 | 0.457 | -0.1390 | Yes |
| 12 | 103221781 | LOC103221781 |  |  | 18345 | 0.456 | -0.0881 | Yes |
| 13 | 103221779 | LOC103221779 |  |  | 18522 | 0.438 | -0.0465 | Yes |
| 14 | 119623524 | LOC119623524 |  |  | 18782 | 0.412 | -0.0114 | Yes |
| 15 | 103221783 | LOC103221783 |  |  | 19328 | 0.359 | 0.0048 | Yes |
| 16 | 103221689 | MHC-DMB |  |  | 19441 | 0.348 | 0.0391 | Yes |
| 17 | 103221792 | LOC103221792 |  |  | 19483 | 0.341 | 0.0758 | Yes |
| 18 | 103247148 | LOC103247148 |  |  | 19749 | 0.309 | 0.0989 | Yes |
| 19 | 103244795 | CD74 |  |  | 20593 | 0.173 | 0.0807 | Yes |
| 20 | 103247136 | LOC103247136 |  |  | 22040 | 0.000 | 0.0161 | Yes |
| 21 | 103221684 | MHC-DOA |  |  | 22082 | 0.000 | 0.0143 | Yes |
Table: GSEA details
[plain text format]

  

Fig 2: ANTIGEN\_PROCESSING\_AND\_PRESENTATION(GO:0019882)
  
Blue-Pink O' Gram in the Space of the Analyzed GeneSet

  

Fig 3: ANTIGEN\_PROCESSING\_AND\_PRESENTATION(GO:0019882): Random ES distribution
  
Gene set null distribution of ES for
 **ANTIGEN\_PROCESSING\_AND\_PRESENTATION(GO:0019882)**
